# Voltage-gated sodium channel as a target for metastatic risk reduction with re-purposed drugs

**DOI:** 10.12688/f1000research.6789.1

**Published:** 2015-07-22

**Authors:** Tomas Koltai

**Affiliations:** 1 Centro de Diagnóstico y Tratamiento de la Obra Social del Personal de la Industria de la Alimentación, Talar, Buenos Aires, C1122AAL, Argentina

**Keywords:** Voltage-gated sodium channels, cancer, phenytoin, flunarizine, repurposed drugs, metastasis

## Abstract

**Objective:** To determine the exact role of sodium channel proteins in migration, invasion and metastasis and understand the possible anti-invasion and anti-metastatic activity of repurposed drugs with voltage gated sodium channel blocking properties.

**Material and methods:** A review of the published medical literature was performed searching for pharmaceuticals used in daily practice, with inhibitory activity on voltage gated sodium channels. For every drug found, the literature was reviewed in order to define if it may act against cancer cells as an anti-invasion and anti-metastatic agent and if it was tested with this purpose in the experimental and clinical settings.

**Results:** The following pharmaceuticals that fulfill the above mentioned effects, were found: phenytoin, carbamazepine, valproate, lamotrigine, ranolazine, resveratrol, ropivacaine, lidocaine, mexiletine, flunarizine, and riluzole. Each of them are independently described and analyzed.

**Conclusions: **The above mentioned pharmaceuticals have shown anti-metastatic and anti-invasion activity and many of them deserve to be tested in well-planned clinical trials as adjunct therapies for solid tumors and as anti-metastatic agents. Antiepileptic drugs like phenytoin, carbamazepine and valproate and the vasodilator flunarizine emerged as particularly useful for anti-metastatic purposes.

## Introduction

The capacity to metastasize is one of the hallmarks of cancer ^1^ and usually death due to cancer is not caused by the primary tumor but rather by the metastatic spread ^2^. The lack of an effective therapy in prevention of metastasis results in a high mortality rate in oncology. So it seems reasonable that if the risk of metastasis can be reduced, the outlook of cancer patients may significantly improve survival and quality of life. Solving the metastasis problem is solving the cancer problem ^3^.

Many natural products, like genistein ^4^, resveratrol ^5^ and curcumin ^6, 7^ have shown interesting anti-metastasis activity. The same effect has been observed with older pharmaceuticals like aspirin ^8^, not-as-old pharmaceuticals such as celecoxib ^6, 9^; new pharmaceuticals like ticagrelor ^10^, as well as with more sophisticated molecules like dasatinib and ponatinib ^6^ or ultrasophisticated drugs, like polymeric plerixafor ^11^.

Many other compounds have also been identified as possessing anti-metastatic effects, including increases in NO ^12^, cimetidine, doxycycline, heparin and low molecular heparins, and metapristone ^13^.

High creativity has been employed in the search for anti-metastatic compounds. For example, Ardiani *et al.* developed a vaccine-based immunotherapy to enhance CD4 and CD8 T lymphocyte activity against Twist ^14^. Twist is a transcription factor involved in invasion and metastasis.

Many known pharmaceuticals that are, or were, in use for other purposes than cancer treatment are demonstrating anti-metastatic activity. This is the case for thiobendazole, which is an antifungal, anti-parasitic drug that has been used in medical practice for over 40 years, but which also shows anti-migratory and apoptosis-inducing activity ^15^. The introduction of Food and Drug Administration (FDA)-approved products which are used for a purpose different for which it was originally approved is called repurposing of a drug.

Many new drugs are being introduced in the area of anti-metastatic activity. One such example, zoledronic acid ^16^ is a biphosphonate that decreases bone metastasis. Denosumab ^17^ is another example. It is a monoclonal antibody directed against the receptor activator of nuclear factor kappa B ligand (RANKL) that diminishes the number of circulating cancer cells and prevents bone metastasis. It is in Phase II clinical trials and has the advantage of subcutaneous administration, while zoledronic acid requires intravenous route (for further information on these compounds, see clinical trials NCT01952054, NCT01951586, NCT02129699) ^18^.

Metastasis is a multi-step development. The different steps in the metastatic cascade can be targeted with a combination of drugs against each step. Migration and invasion are necessary steps for the metastatic cascade. There is no metastasis without prior migration of malignant cells, so that if migration and invasion are blocked, metastasis should not occur.

**Figure 1.**
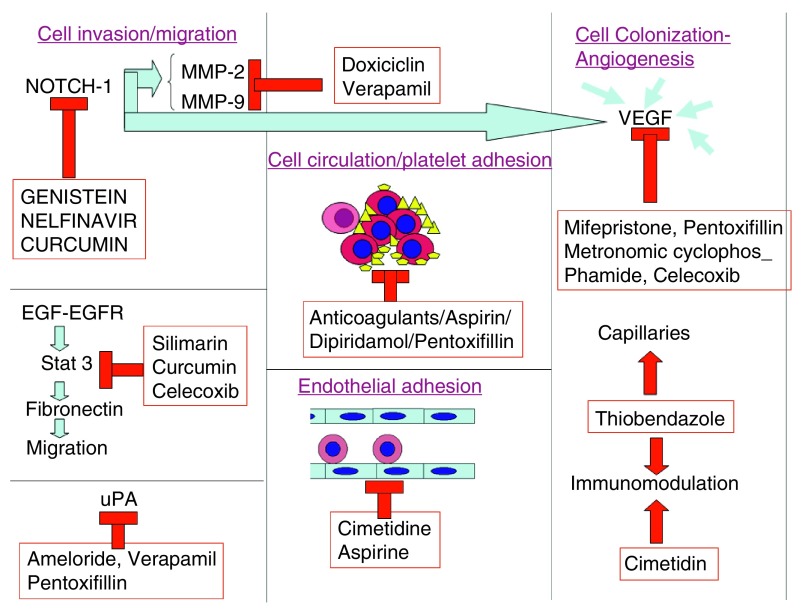
Repurposed drugs acting at different levels of the metastatic cascade. (uPA: urinary plasminogen activator).

Invasion is the first step in metastasis, and in a very simplified view, it can be divided into three stages (Shown schematically in Figure 1):

1. Translocation of cells across extracellular matrix barriers
2. Degradation of matrix proteins by specific proteases
3. Cell migration

## Voltage-gated sodium channels

Neurons and muscle cells (and excitable tissues in general) express voltage-gated sodium channel (VGSC) proteins; tumor cells may also express these proteins. VGSCs are important players in migration and invasion as it will be described in this manuscript.

Sodium channels were first described by Hodgkin and Huxley in 1952 and knowledge about structure and physiology of VGSCs are mainly the result of seminal investigations developed by William Catterall ^19^.

Sodium channels are glycosylated transmembrane proteins that form passages in the cell membrane for the penetration of sodium into the intracellular space according to their electrical gradients. Voltage-gated sodium channels (also known as VGSCs or ‘NaV’ channels) refers to the mechanism that triggers these proteins to allow sodium movement across the membrane.

There are nine known VGSCs (NaV1.1 to Nav1.9) that are members of the superfamily of VGCSs. NaV1.1, 1.2, 1.3 and 1.6 are found in the central nervous system. NaV1.4 is found in muscle and NaV1.5 in cardiac muscle ^20^.

VGSC is formed by a large subunit (α) and other smaller subunits (β). The α subunit is the core of the channel and is fully functional by itself, even without the presence of β subunits ^19– 21^.

**Figure 2.**
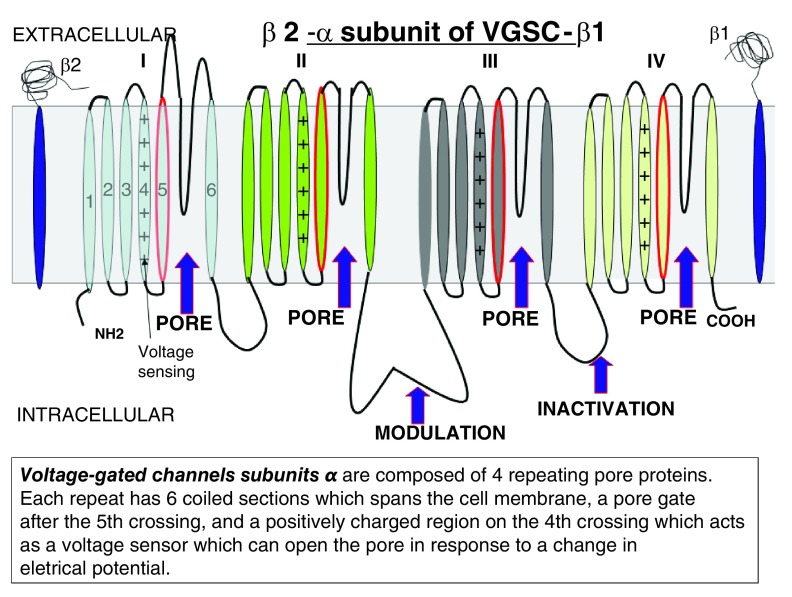
An idealized drawing of α and β units of VGSCs.

**Figure 3.**
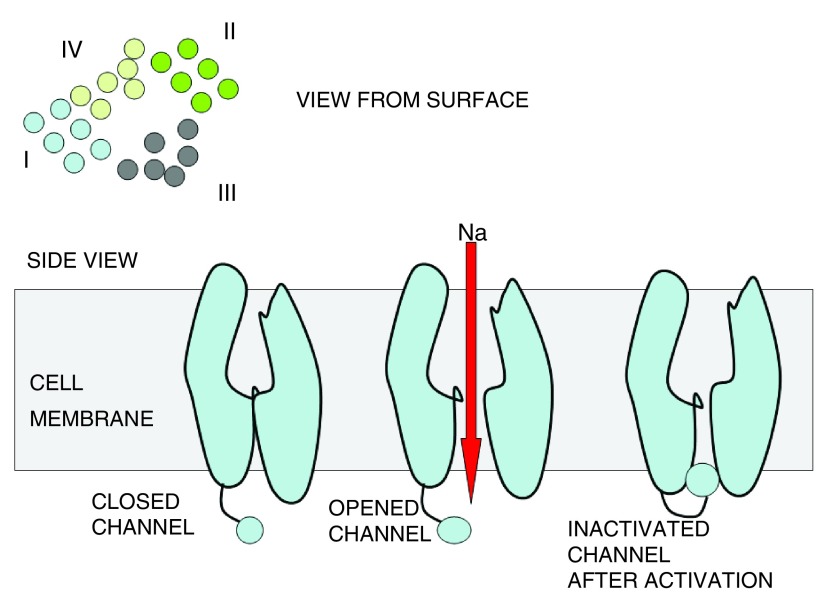
Surface and side view of VGSC.

When a cell expresses VGSC α subunits, this means that it is capable of conducting sodium into the cell. The structure of VGSC can be seen in Figure 2 and Figure 3. VGSCs modulate the exchange of Na+ across the cell membrane and the inflow of this electrolyte spikes the action potential in excitable tissues ^22^.

It is well known that expression of VGSCs appears in cancer cells where it is not expressed in their normal counterparts, and plays a significant role in disease progression. Table 1 shows examples of the cancer tissues in which dysregulated expression of VGSCs were identified and the role they play.

**Table 1.** VGSC functional over-expression in different cancer tissues.

Cancer	Reference	Findings
Human breast cancer	Fraser, 2005 ^23^	VGSC (neonatal isoform of NaV1.5) was significantly upregulated in metastatic cells. VGSC activity increased endocytosis, migration and invasion
Non small-cell lung cáncer (NSCLC)	Roger, 2007 ^24^	Strongly metastatic cell lines have functional VGSCs while normal cells do not have it. Inhibition of channels with tetrodotoxin (TTX) reduced invasivenes by 50%.
Small cell lung cáncer (SCLC)	Blandino, 1995 ^25^	VGSCs are over-expressed in SCLC.
Cervical cancer cells	Diaz, 2007 ^26^	Na _v_1.2, Na _v_1.4, Na _v_1.6, and Na _v_1.7 transcripts were detected in cervical cancer cell specimens.
Prostate cancer	Bennett, 2004 ^27^	VGSC expression increases with invasion capacity that can be blocked with TTX. Increased VGSC expression is enough to increase invasive phenotype.
Metastatic ovarian cancer cells	Gao, 2010 ^28^	Highly metastatic ovarian cells showed significantly elevated expression of Nav1.2, Nav1.4, Nav1.5 and Nav1.7. TTX reduced migration and invasion around 50%.
Human colon cancer cells	House, 2010 ^29^	*SCN5A,* the gene coding for α sububit of VGSC is a regulator of the invasive phenotype.
T lymphocytes (Jurkat cells)	Fraser, 2004 ^30^	Jurkat cells express VGSC and this protein has an important role in invasiveness of these cells.
Pancreatic cancer cells	Sato K, 1994 ^31^	MIA-PaCa-2 and CAV cells were tested *in vitro* and *in vivo* with phenytoin (PHEN). Both cell lines showed growth inhibition in a dose dependable manner. This might be due to VGSC overexpression according to our criteria (the authors think that this was due to calcium channel blocking).
Mesothelial neoplastic cells	Fulgenzi, 2006 ^32^	Express VGSCs, particularly NaV1.2, and NaV1.6, and NaV1.7. TTX decreased cell motility and migration.

Targeting these channels may represent a legitimate way of reducing or blocking the metastatic process.

The role of sodium channel in invasion, metastasis and carcinogenesis is insufficiently known.

## Sodium channel proteins and cancer

In 1995, Grimes *et al.* ^33^ investigated the differential electrophysiological characteristics of VGSCs in two different rodent prostate cancer cell lines: the Mat-Ly-Lu cell line, which is a highly metastatic line (more than 90% of metastasis to lung and lymph nodes under experimental conditions) and the AT-2 cell line with a much lower metastatic potential (less than 10% chance of developing metastasis in experimental conditions). They found fundamental differences in electrophysiological features between these two cell lines which displayed a direct relationship with *in vitro* invasiveness. Sodium inward currents were detected only in the Mat-Ly-Lu cell line and inhibition of VGSC protein with Tetrodotoxin (TTX; a powerful inhibitor of VGSCs) significantly reduced the capacity for invasion (mean reduction 33%). On the other hand, TTX showed no effect on invasion of AT-2 cell lines. The TTX-induced reduction of invasion showed a direct correlation with the amount of cells expressing VGSC in the culture.

No fundamental differences in the potassium channels were found between the two cell lines, except for a lower density of potassium channels in the Mat-Ly-Lu cell line. The authors concluded that ion channels may be involved in malignant cell behavior and that VGSCs could play a role in the metastatic process.

In 1997, Laniado *et al.* ^34^ investigated the presence of VGSC in human prostate cell lines. As in the Grimes research they used two different cell lines: one with a low metastatic potential: the LN-Cap cell line which is androgen dependent and expresses prostate-specific antigen, and the PC-3 line which is more malignant, does not express prostate-specific antigen and exhibits a high rate of metastatic potential.

As in the work by Grimes *et al.*, they found that PC-3, the more malignant cell line, expressed VGSC protein and that inhibition of this channel protein with TTX reduced invasion in a significant way. LN-Cap cells did not express VGSC.

One of the conclusions reached by the authors was that cancer cells expressing functional VGSC had a selective advantage regarding migration and distant metastasis. In the case of both humans and rodents, not all cells in the highly malignant cell cultures showed the presence of the VGSC protein. For example, in PC-3 cell culture only 10% of cells expressed a functional VGSC protein. This is the reason why the authors consider these cells as a clonal evolution that gives pro-tumor and pro-invasive advantages.

The correlation between VGSC protein expression and invasiveness in human and rat prostate cancer cells was confirmed by Smith *et al.* ^35^ by comparing seven lines of rat prostate carcinoma cells with different metastatic ability, and nine human prostate carcinoma cell lines. In general, invading capacity of the basement membrane and metastatic ability showed a positive correlation with the percentage of cells expressing VGSC. But this positive correlation between percentage of cells expressing VGSCs and the percentage of cells being invasive occurred only up to 27% of the cells being invasive in the rat series and up to 12% of cells being invasive in the human series. Authors suggested that these discrepancies may be due to the necessity of other factors for invasive capability besides VGSC presence; i.e. this protein may represent a prerequisite for the invasive phenotype but other requirements must also be achieved for a full-blown invasive phenotype. Fraser *et al.* ^36^ determined the key role played by VGSCs in prostate cancer cells in invasion and motility and showed that TTX and phenytoin (PHEN) that are known VGSC blockers, decreased motility and invasiveness while channel openers increased motility. However, the increased invasion capacity in VGSC-expressing cancer cells is not limited to prostate cancers. The same features were found in breast cancer cell lines MCF-7 (estrogen receptor positive), MDA-MB-231 and MDA-MB-468 (both estrogen receptor negative).

Baciotglu *et al.* ^38^ when experimenting on a rat model of induced breast cancer showed the importance of inhibiting VGSCs in order to inhibit antioxidant response. They observed a survival improvement in rats treated with a VGSC blocker.

**Figure 4.**
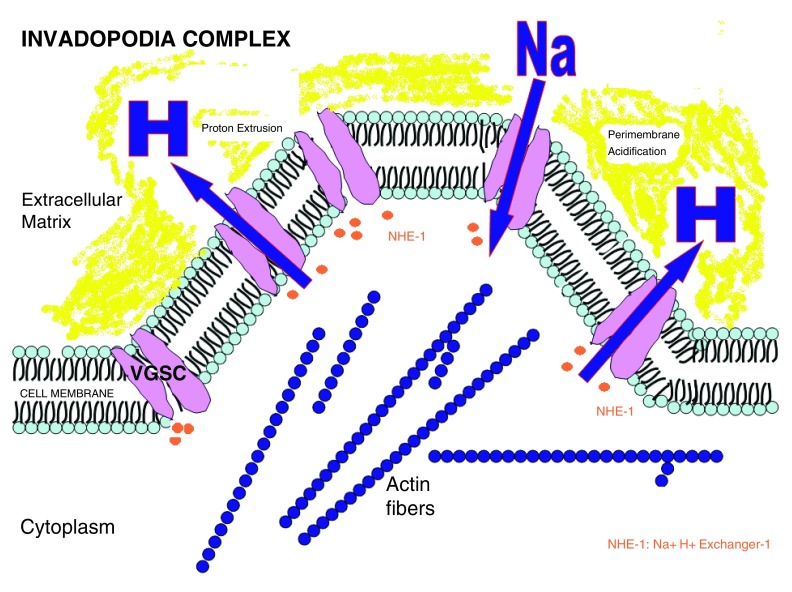
Proton extrusion through VGSC and NHE-1 (sodium hydrogen exchanger-1) produces acidification of extracellular matrix surrounding the cellular membrane (yellow area in the figure). (Brisson 2013 ^39^; Gillet 2009 ^40^). Acidification activates cathepsine degradation of the extracellular matrix.

**Figure 5.**
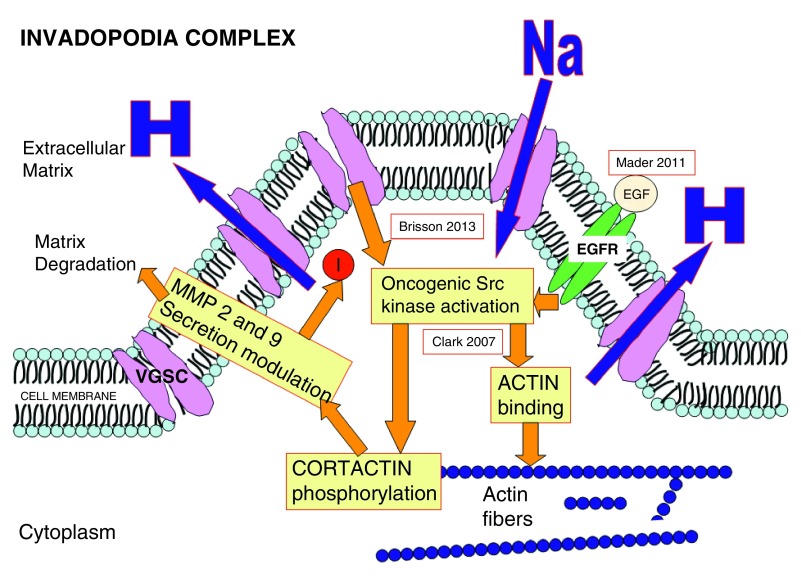
VGSCs. The second mechanism of action is through activation of Src which increases MMP-2 and MMP-9 secretion and activity through phosphorylation of Cortactin. It is postulated that there is a feedback loop starting with MMPs products which induces the development of new invadopodia (Red circle around I). (This figure has been constructed based on references Gillet 2009 ^40^, Brisson 2013 ^133^, Clark 2007 ^42^ and Mader 2011 ^41^).

An important location of VGSCs in cancer cells is in a cellular region directly involved in migration and invasion: the invadopodia. Invadopodias are protrusions of the plasma membrane, rich in actin that are strongly related to degradation of the extracellular matrix (ECM). Figure 4 and Figure 5 summarize how invadopodia works and the relation between VGSC and invadopodia.

According to Brisson *et al.* ^39^, NaV 1.5 Na+ channels regulate the NHE-1 exchanger protein that increases proton extrusion with extracellular matrix acidification that promotes invasion and migration through activity of cystein cathepsines and degradation of extracellular matrix ^40^.

A second mechanism of invasion promotion was described by Mader *et al.* ^41^ through the EGFR-Scr-cortactin pathway. Src is activated by VGSCs and Src phosphorylates cortactin. Cortactin is involved in MMP-9 and MMP-2 upregulation and secretion as can be seen in Figure 5. These events lead to matrix degradation, an integral step in cancer cell invasion ^42^.

There are nine different VGSC α subunits and four different β subunits. The expression of these subunits may vary in the different tumor cells ^21^. For example, NaV 1.5 is overexpressed in astrocytoma, breast and colon cancer. NaV 1.7 is found in breast, prostate and non small-cell lung cancer (NSCLC) and NaV 1.6 in cervical and prostate cancer. This suggests that the α subunits seem to be tissue specific.

The main players in the invadopodia complex, besides the VGSCs are Src kinase, cortactin and Rho-A GTPase. The exact relation between these players is not fully known and needs further research. (For further reading on invadopodia and cortactin, see references 43, 44).

**Figure 6.**
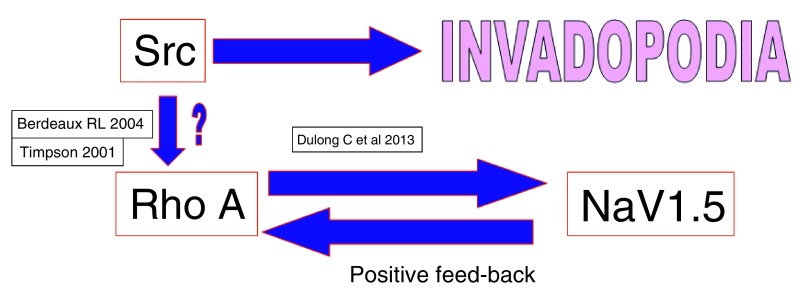
Tarone *et al.* ^46^ in 1985 reported the relation between the oncogenic Src and promotion of invadopodia. Berdeaux *et al.* ^47^ reported that the small molecule GTPase Rho A activity is under control of oncogenic Src and localizes in the invadopodia complex and Durlong *et al.* ^48^ (2013) showed that Rho-A regulates the expression and activity of NaV1.5 and found a positive feedback between NaV1.5 and Rho A in breast cancer cells. According to Timpson *et al.* ^49^, cooperation between mutant p53 and oncogenic Ras activates Rho-A.

One possible relation between invadopodia-Src-VGSCs is described in Figure 6.

**Figure 7.**
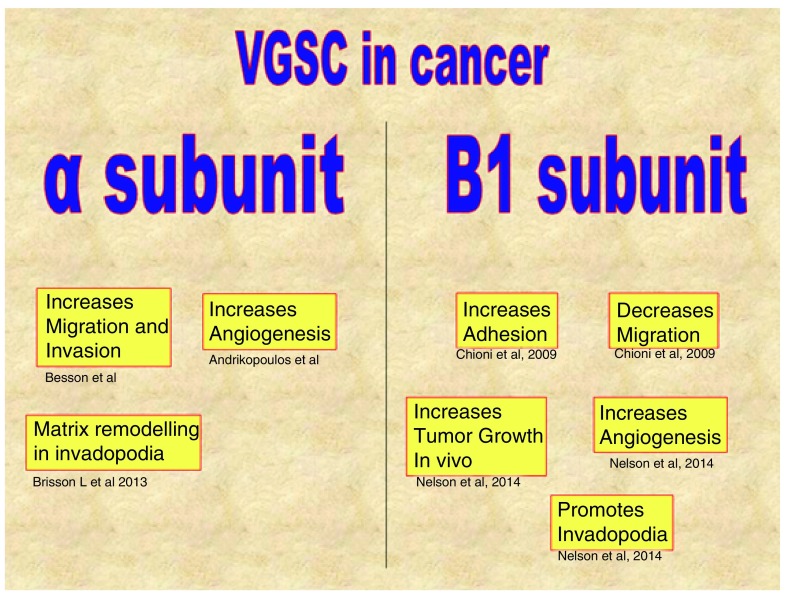
Activities of alpha and beta-1 subunits of VGSCs in cancer ^131– 133^.

Onganer and Djamgoz ^45^ proposed the hypothesis that VGSC upregulation enhances the metastatic phenotype by enhancing endocytic membrane activity in SCLC.

Andrikopoulus *et al.* ^50^ have demonstrated that VGSCs have pro-angiogenic functions by significantly increasing vascular endothelial growth factor (VEGF) signaling in endothelial cells. Endothelial cells express NaV1.5 and NaV1.7. TTX blocks, and NaV1.5 RNAi decreases endothelial cell proliferation and tubular differentiation that are essential steps in the angiogenesis process.

The important implications of VGSCs in cancer progression and invasion led Litan and Langhans ^51^ to express that cancer is a channelopathy (For further reading on structure and functions of VGSC see reference 52).

## Material and methods

A search was performed in the medical literature to find pharmaceuticals already in use for other purposes than cancer, that as an off-target effect could inhibit VSGCs and to determine if these pharmaceuticals can actually decrease migration, invasion and metastatic potential of cancer. A Pubmed advanced search retrieved 50519 articles under the search condition “voltage-gated sodium channel blocker” during the period of 1981–2015.

The articles that considered drugs that were not in clinical use or FDA-approved were not included in this study, with the exception of resveratrol and natural polyphenols.

The following drugs fulfilling these criteria were found: phenytoin, carbamazepine, lamotrigine valproate, ranolazine, resveratrol, ropivacaine, lidocaine, mexiletine, flunarizine, and riluzole.

A new search was performed in Pubmed for each of the above listed pharmaceuticals with two search criteria: 1) the drug and 2) the term cancer. The period considered was from 1962 to the current time.

Those VGSC blocking drugs that exhibited anti-cancer activity based mainly by other mechanisms are only briefly mentioned; valproic acid and lamotrigine probably act against cancer by histone deacetylase inhibition and riluzole’s anti-cancer mechanism is probably related to the glutamatergic pathway.

Tetrodotoxin is also analyzed in spite of the fact that it is not in clinical use, because it is the traditional model molecule of VGSC blocking, against which other drugs are comparatively tested in the experimental setting.

## Results

### Tetrodotoxin (TTX)

Many biological toxins like those found in scorpions and sea anemones develop their toxicity by introducing modifications to the properties of VGSCs ^52^. This toxicity can be achieved by inactivation of VGSCs (as in the case of TTX) or on the contrary by persistent activation of the channel (in the case of veratridine, acinitine and many others).

TTX is a powerful biological neurotoxin found in fishes of the Tetraodontiformes order and certain symbiotic bacteria. TTX binds to VGSCs and blocks its activity, mainly in the nervous system. It is used as a biotoxin for defensive or predatory purposes. TTX binds to the extracellular portion of VGSC, disabling the function of the ion channel and results in a very poisonous effect producing death through respiratory paralysis ^22^.

Due to its high toxicity it is not used as a therapeutic agent, but TTX has been very useful in the experimental setting for the study of VGSCs physiology.

### Phenytoin (Diphenylhydantoin; PHEN)

PHEN is an anticonvulsant that has been identified as a sodium channel blocker ^53, 54^ which has been held responsible for inducing lymphoma, pseudolymphoma, hematological malignancies and other cancers in patients under chronic treatment ^55^. This carcinogenic effect of phenytoin was not confirmed in large epidemiological studies ^56^. PHEN diminishes cell mediated immunity ^57^.

Vernillo *et al.* in 1990 ^58^ found that phenytoin inhibited bone resorption in rat osteosarcoma cells through significant reduction of collagenase and gelatinase activities. But Dyce *et al.* ^59^ did not find evidence of PHEN’s gelatinase inhibitory activity in B16 melanoma cells *in vitro*. This may be evidence of tissue-specific activity which has not been investigated any further. Dyce *et al.* did not find important anti-metastatic activity either in a melanoma tail injection model in mice. But when the data of this publication is examined in detail, it seems that the anti-metastatic activity is not so low as mentioned by the authors: they found that after injection of tumor cells in the mice protected with PHEN, the animal developed mean pulmonary colonies 4.6 +/- 3.1 but when the mice received no PHEN, developed. 10.2 +/- 9.9 colonies. Beyond any statistical analysis the difference seems important.

Yang *et al.* ^60^ found that NaV 1.5 was over-expressed in breast cancer cells with high metastatic potential, and the anticonvulsivant PHEN had the ability to reduce migration and invasion at clinically achievable concentrations in MDA-MB-231 cells (which are strongly metastatic) and showed no effects on MCF-7 cells with low metastatic potential.

PHEN blocks Na ^+^ channels and has a high affinity for VGSCs in the inactivated state of the channel ^61^. Compared with verapamil, lidocaine and carbamacepine, PHEN had an intermediate potency between verapamil and lidocaine, being verapamil the strongest inhibitor and carbamazepine the weakest.

Abdul *et al.* ^62^ studied the effect of four anticonvulsants (PHEN, carbamazepine, valproate and ethosuxinide) on the secretion of prostate specific antigen and interleukin-6 in different human prostate cancer cell lines. PHEN and carbamazepine inhibited the secretion of both.

Fadiel *et al.* ^63^ found that PHEN is a strong estrogen receptor α antagonist at clinically achievable concentrations and at the same time is a weak agonist.

**Table 2.** Summary of PHEN’s activity against cancer and metastasis.

Reference	Findings
Nelson, 2015 ^64^	PHEN at clinically achievable concentration reduces breast cancer growth, invasion and metastasis *in vivo* in a xenograft model.
Yang M, 2012 ^60^	At clinically achievable concentration PHEN inhibited migration and invasion of highly metastatic breast cancer MDA-MB-231 cell line, and had no effect on MCF-7 cell line which has a low metastatic potential and do not express VGSCs.
Lee Ts, 2010 ^65^	DPTH-N10 a phenytoin derivate drug, inhibits proliferation of COLO 205 colon cancer cell line.
Lyu Y, 2008 ^66^	DPTH-N10 a phenytoin derivate drug, shows strong anti-angiogenic activity. Inhibits HUVEC proliferation and capillary like tube formation.
Sikes, 2003 ^67^	New VGSC blockers based on PHEN, showed increased inhibition of prostate cancer growth.
Anderson, 2003 ^68^	Developed new VGSC blockers based on the PHEN binding site study. These new VGSC blockers showed potent inhibition of prostate cancer cells growth (Androgen independent PC3 line).
Fraser, 2003 ^36^	PHEN decreased motility of prostate cancer cells.
Abdul, 2001 ^62^	PHEN and carbamazepine decreased PSA secretion in human prostate carcinoma cell lines.
Lobert, 1999 ^69^	PHEN has inhibitory effects on microtubule assembly and has additive effects with vinblastine.
Kawamura, 1996 ^70^	PHEN potentiates vinblastine citotoxicity.
Sato K, 1994 ^31^	PHEN decreased growth of MIA PaCa-2 (pancreatic cancer cells).
Lang DG, 1993 ^71^	PHEN, Carbamazepine and lamotrigine are inhibitors of sodium channels and reduces glutamate release in rat neuroblastoma cells.
Tittle, 1992 ^72^	PHEN decreased growth in six murine tumor cell lines of lymphoid origin.

There is an undesired side effect of PHEN that may represent a drawback for its use in cancer: immunological depression ^73– 76^. This is an issue that deserves further research. Finally it has to be mentioned that PHEN interacts with many other pharmaceuticals, particularly those usually employed in chemotherapy.

In summary, the main activities developed by PHEN in relation with cancer are:

VGSC blocking, microtubule polymerization blocking, immunosuppression, calcium channel blocking and enhancement of vinblastine cytotoxicity.

### Carbamazepine

Carbamazepine is a sodium channel blocker, pro-autophagy agent and histone deacetylase inhibitor that has been in use since 1962 for the treatment of seizures, neuropathic pain and bipolar disorders and has shown interesting anti-metastatic potential in the experimental setting ^77^. Studies have also insinuated preventative effects in prostate cancer ^78^.

Carbamazepine induces Her2 protein degradation through the proteosome without modifying its production ^79^. This activity seems to be related to histone deacetylase inhibition rather than VGSC blocking. Growth inhibition in estrogen-receptor positive breast cancer cell lines seems probably a histone deacetylase inhibitor effect ^80^.

Oxcarbazepine, a molecule related to carbamazepine is also a sodium channel blocker ^81^ and a potassium channel blocker, but it has not been investigated for cancer.

The anti-cancer mechanisms shown by carbamazepine are in summary:

1) VGSC blocker as anti-metastatic ^77^.
2) Histone deacetylase inhibition ^82^.
3) Her2 degradation by proteasome ^79^.

### Valproic acid (VAL)

An anticonvulsivant drug that exerts multiple actions related to anti-cancer effects: calcium channel blocker, VGSC blocker, inhibition of histone deacetylase, potentiation of inhibitory activity of GABA, decreases angiogenesis, interferes with MAP kinase pathways and the β catenin-Wnt pathway ^83^. Val is being tested in various clinical trials in leukemias and solid tumors ^84^. Most of the anti-tumor activities of VAL seem to be related to the inhibition of histone deacetylase rather than VGSC blocking and further discussion goes beyond the scope of this review.

### Ranolazine

Ranolazine, (Ranexa) is an antiarrhythmic drug indicated for the treatment of chronic angina that was first approved by FDA in 2006. Common side effects are dizziness, constipation, headache and nausea ^85^.

Ranolazine inhibits the late inward sodium current in heart muscle, so that it works as a sodium channel inhibitor. Ranolazine is metabolized by the CYP3A enzyme.

Driffort *et al.* ^86^ demonstrated that ranolazine inhibition of NaV1.5 reduced breast cancer cells invasiveness *in vivo* and *in vitro* using the highly invasive MDA-MB-231 breast cancer cell line. This drug also efficiently decreased the activity of the embryonic/neonatal isoform of NaV1.5 (the active isoform usually found in human breast cancer cells). Ranolazine did not change the viability of the cell. It also decreased the pro-invasive morphology of MDA-MB-231 breast cancer cells. They also demonstrated that injection of cancer cells through the tail vein of nude mice at non-toxic doses achieved a significant reduction in metastatic colonization.

### Resveratrol and other polyphenols

Certain biologically active natural phenols like resveratrol and genistein have shown effects on VGSCs, increasing hyperpolarized potentials during steady state inactivation ^88^.

Resveratrol’s inhibitory effects on VGSC has consequences for the behaviour of metastatic cells. Fraser *et al.* ^89^ showed that resveratrol significantly decreased lateral and transversal motility and invasion capacity of rat prostate cancer cells (MAT-Ly-Lu cells), without changes in cellular viability. They also found that resveratrol inhibited VGSC in a dose-dependent manner and using TTX with resveratrol did not increase VGSC inhibition nor metastatic cell behavior. Resveratrol also inhibits epithelial sodium channels ^90^.

 Resveratrol is not the only polyphenol with VGSC blocking activity: quercetin and catechin showed similar effects ^91^ in ventricular myocytes and genistein in rat cervical ganglia ^92^ and nociceptive neurons ^93^.

### Gabapentin

Gabapentin is used for the treatment of pain and decreases expression of NaV1.7 and ERK-1/ERK-2 in ganglion neurons ^94, 95^ and expression of NaV1.2 ^96^. We found no publications about gabapentin as a possible anti-metastasis or anti-invasion treatment.

### Riluzole

Riluzole is a drug used for amyotrophic lateral sclerosis and it is a known sodium channel blocker. This effect was demonstrated in human prostate cancer cell lines ^97^. Most likely, the anticancer activity of riluzole is mainly related to other anticancer characteristics of this drug like downregulation of the glutamatergic pathway ^98^.

### Flunarizine

Flunarizine is a calcium channel blocker with a long plasma half-life, used in migraine prevention, vertigo and adjuvant treatment of epilepsy, but has shown important activity as a VGSC blocker ^99– 101^. It binds calmodulin.

At low temperatures (22 degrees) flunarizine potentiate the binding of phenytoin to VGSC ^102^.

Flunarizine has shown anti-cancer activities in lymphoma and multiple myeloma ^103^, and leukaemia ^104^, but these anti-cancer activities were apparently related to induction of apoptosis, which is not a consequence of VGSC blockage. On melanoma cells, flunarizine showed decreased motility and invasion *in vitro* ^105, 106^ *.* Flunarizine inhibited migration and phagocytosis in B16 melanoma cells and M5076 macrophage-like cancer cells ^107^.

According to data found in medical literature we may consider anti-cancer activities of flunarizine in the following way:

a) Activities dependent on VGSC blocking: decreased motility and invasion ^105– 107^.
b) Activities dependent on calcium channel blocking: vasodilatation and increased concentration of chemotherapeutic drugs in tumor tissues ^108– 110^, and increased radiosensitivity due to better oxygen delivery to anoxic areas of the tumor ^111^.
c) WNT inhibition ^103^.
d) Inhibition of lymphangiogenesis ^112^.
e) Increase of melphalan`s citotoxicity in resistant ovarian cancer cells ^113^ and in rhabdomyosarcoma ^114^.
f) Positive modulation of doxorubicin in multidrug resistant phenotype colon adenocarcinoma cells ^115^.
g) Decreased blood viscosity improving oxygen delivery to the tumor ^116^.
h) Other anti-tumor activities: apoptosis and growth rate inhibition ^103, 104, 117^.

Flunarizine has not been tested in cancer trials. The fact that it can significantly reduce motility in melanoma cells which is a highly metastasizing tumor and is also an inhibitor of lymphangiogenesis, makes it an interesting adjuvant therapy that deserves further research. The possible synergy with phenytoin is also an issue that should be explored.

However, flunarizine has also shown cytoprotective effects in certain tissues (auditory cells) against cisplatin ^118^ and flunarizine may induce Nrf-2 overexpression that confers resistance to chemotherapy in some tumors like Her2 positive breast cancer ^119^.

### Local anaesthetics

Local anaesthetics eliminate pain through VGSC blocking on nociceptive neurones.

Local anaesthetics like lidocaine have shown interesting anti-cancer effects in various cancer cells. Lidocaine is a VGSC blocker. The mechanisms involved in decreased proliferation seems related to the inhibitory actions of local anaesthetics on EGFR ^120^ rather than VGSC blocking. Inhibition of invasion found in cancer cells treated with lidocaine (HT1080, HOS, and RPMI-7951) by Mammoto *et al.* was attributed by the authors to shedding of the extracellular domain of heparin binding epidermal growth factor-like growth factor and not to VGSC blocking ^121^.

Baptista-Hon *et al.* described a decrease in metastatic potential of colon cancer cells (SW620 cells) by ropivacaine and decrease of Nav1.5 function (adult and neonatal isoforms) ^122^.

Piegeler *et al*., 2012 identified decreased Src activity produced by amide-linked anaesthetics as an independent mechanism of migration and invasion decrease ^123^.

### Other drugs

Other drugs that have shown significant VGSC blocking activity and may have activity in the fight against migration, invasion and metastasis are: fluoxetin blocks NaV1.5 ^124^, and mexiletine ^125^.

Intravenous propofol has been recognized as an anti-invasion drug in HeLa, HT1080, HOS and RPMI-75 cells by decreasing actin stress-fiber formation and focal adhesion inhibition, but this drug is not a VGSC blocker and the probable mechanism is through Rho-A modulation ^126^.

All of the drugs we have mentioned are low cost pharmaceuticals, have predictable and well known side effects, and therefore they are adequate candidates for further clinical trials.

## Discussion

Functionally active VGSCs are expressed in many metastatic cancer cells. This functional expression is an integral element of the metastatic process in many different solid tumors.

The essential role of this protein in invadopodia has been established, so VGSCs became a legitimate target to decrease migration, invasion and metastasis. Repurposed drugs like anticonvulsants, (phenytoin in particular) have shown interesting anti-invasion effects.

Carbamazepine’s ability to induce Her2 protein degradation should be considered an interesting association to trastuzumab.

Targeting VGSCs may act in synergy with anti-angiogenic treatments and with other chemotherapeutic drugs like vinblastine.

On the other hand in the review by Besson *et al.* ^127^ there is a very important remark: VGSC is also present in macrophages and cells related with the immunologic system, so that disrupting VGSC`s activity may deteriorate also anti-tumor immunologic mechanisms.

Flunarizine represents a particularly interesting molecule because it may attack cancer from four different angles: invasion and migration through VGSC blocking, WNT pathway down-regulation, decreased lymphangiogenesis and better oxygenation of hypoxic areas which permits a better arrival of chemotherapeutic drugs and increased sensitivity to radiation. It has never been tested in clinical trials for cancer treatment.

## Future directions

New VGSCs blockers are under research. Sikes *et al.* ^67^ developed new blockers based on the phenytoin binding site to VGSC. They found compounds with enhanced activity in VGSC blocking and antitumor activity against human prostate cancer cells.

The association of two or more VGSC blockers may show synergistic enhanced anti-metastatic activity. Nerve growth factor (NGF) increases the number of VGSCs ^129^; tanezumab, a new NGF inhibitor diminishes the amount of VGSCs ^128^, so we may assume that tanezumab may develop synergistic activity with VGSC blockers. Tanezumab has not been tested in cancer and we think it deserves more research because NGF is also an anti-apoptotic protein ^130^.

Stettner *et al.* ^134^ found that men over 50 years of age may be benefited with the use of anticonvulsivants, regarding prostate cancer prevention because they observed lower PSA levels compared with control groups. They also described a ranking of this preventive activity: valproic acid>levetiracetam>carbamazepine/oxcarbazepine>lamotrigine. The authors also observed synergy between these drugs.

## Conclusions

Repurposed VGSC blocker drugs, particularly phenytoin, flunarizine and polyphenols, deserve clinical trials as complementary treatment to decrease the metastatic risk.
