# Supplementary material for: Voltage-gated sodium channel as a target for metastatic risk reduction with re-purposed drugs
Source: F1000Res. 2015 Jul 22;4:297. [Version 1] doi: 10.12688/f1000research.6789.1 (PMC4920216; doi:10.12688/f1000research.6789.1)
Supplement: Peer review history

# Peer review: Referee response for version 1

**DOI:** 10.5256/f1000research.7293.r14301

**Published:** 2016-06-22

**Authors:** Michihiro Mutoh

**Affiliations:** 1 Epidemiology and Prevention Division, Research Center for Cancer Prevention and Screening, National Cancer Center, Chuo-ku, Japan

This manuscript is well summarized with topics. However, I believe some more information needs to be added.

  

1. As small molecules, such as FDA approved drugs, affect the tissue of the whole body, the information of the data from genetic findings, such as NaV1.5 knockout mice data, is desired to be included in the text
2. The idea that selecting the voltage gated sodium channel blocker by repurposing of a drug seems attractive to fight invasion and metastasis of cancer. However, a concrete example for the medical use is not shown in the text. Please provide an idea when a patient could use such a drug.  
3. Oxidative stress contributes to the invasion process. Figures 3 & 4 show that proton exfusion also plays a role in the invadopodia complex. Do the author has an idea to connect oxidative stress and the voltage gated sodium channel? If not, please ignore this comment.
4. Figure 7: “B1 subunit” should be replaced by “b(symbol)1 subunit”.
5. The author describes in the text that the voltage gated sodium channel is overexpressed in cancer tissue, and its blockers could be used as cancer chemoprevention agents. It might be worth writing a review article regarding carcinogenesis and the voltage gated sodium channel blocker in the future.

I have read this submission. I believe that I have an appropriate level of expertise to confirm that it is of an acceptable scientific standard.

# Peer review: Referee response for version 1

**DOI:** 10.5256/f1000research.7293.r13165

**Published:** 2016-05-19

**Authors:** Stephen Waxman

**Affiliations:** 1 Department of Neurology, Yale University, New Haven, CT, USA

This article focuses attention on sodium channels as potential therapeutic targets for metastatic disease.  Much work needs to be done to determine whether agents that act on sodium channels will blunt metastatic activity in a clinically meaningful manner, but a spectrum of sodium channel blockers with only minimal side-effects are already used clinically.  This novel approach thus merits careful study.

 While at first glance it may seem surprising that voltage-gated sodium channels are proposed as molecular targets within (presumably) non-excitable cells, there are many precedents for a role of these channels in controlling effector actions in multiple types of cells that have traditionally been considered non-excitable ^1^.  One example is provided by astrocytes, which express multiple types of sodium channels that are functional within the cell membrane ^2^. These astrocytic sodium channels provide a return pathway for Na ions that facilitates operation of the Na-K/ATPase in these cells ^3^.  Notably, expression of sodium channels within astrocytes is highly dynamic, a phenomenon that is strikingly seen in scarring astrocytes in disorders such as multiple sclerosis (MS) and its models where expression of Nav1.5 is up-regulated ^4^.   Recent evidence indicates that these glial sodium channels participate in the astrocytic response to injury, via a cascade that involves Na influx that activates reverse (Ca-importing) Na/Ca exchange ^5^.  Given the large number of traditionally non-excitable cell-types (including microglia, macrophages, and multiple other cell-types) where expression of voltage-gated sodium channels and a functional role for these channels has been documented (reviewed in Black and Waxman, 2013 ^1^), sodium channels may emerge as therapeutic targets in multiple disorders.

I have read this submission. I believe that I have an appropriate level of expertise to confirm that it is of an acceptable scientific standard.

## References

[1] BlackJAWaxmanSG: Noncanonical roles of voltage-gated sodium channels. *Neuron*.2013;80(2) : 10.1016/j.neuron.2013.09.012 280-91 10.1016/j.neuron.2013.09.012 24139034

[2] SontheimerHBlackJARansomBRWaxmanSG: Ion channels in spinal cord astrocytes in vitro. I. Transient expression of high levels of Na+ and K+ channels. *J Neurophysiol*.1992;68(4) :985-1000 133135810.1152/jn.1992.68.4.985

[3] SontheimerHFernandez-MarquesEUllrichNPappasCAWaxmanSG: Astrocyte Na+ channels are required for maintenance of Na+/K(+)-ATPase activity. *J Neurosci*.1994;14(5 Pt 1) :2464-75 818242210.1523/JNEUROSCI.14-05-02464.1994PMC6577452

[4] BlackJANewcombeJWaxmanSG: Astrocytes within multiple sclerosis lesions upregulate sodium channel Nav1.5. *Brain*.2010;133(Pt 3) : 10.1093/brain/awq003 835-46 10.1093/brain/awq003 20147455

[5] PappalardoLWSamadOABlackJAWaxmanSG: Voltage-gated sodium channel Nav 1.5 contributes to astrogliosis in an in vitro model of glial injury via reverse Na+ /Ca2+ exchange. *Glia*.2014;62(7) : 10.1002/glia.22671 1162-75 10.1002/glia.22671 24740847PMC4060891
